# Clinical Efficacy and Cost-Effectiveness of Imagery Rescripting Only Compared to Imagery Rescripting and Schema Therapy in Adult Patients With PTSD and Comorbid Cluster C Personality Disorder: Study Design of a Randomized Controlled Trial

**DOI:** 10.3389/fpsyt.2021.633614

**Published:** 2021-03-19

**Authors:** Arne van den End, Jack Dekker, Aartjan T. F. Beekman, Inga Aarts, Aishah Snoek, Matthijs Blankers, Chris Vriend, Odile A. van den Heuvel, Kathleen Thomaes

**Affiliations:** ^1^Sinai Centrum, Amstelveen, Netherlands; ^2^Department of Psychiatry, Academic Medical Center, Location Vrije Universiteit Medical Center, Amsterdam, Netherlands; ^3^Arkin Mental Health Care, Amsterdam, Netherlands; ^4^Faculty of Behavioural and Movement Sciences, VU University, Amsterdam, Netherlands; ^5^GGZ inGeest, Amsterdam, Netherlands; ^6^Netherlands Institute of Mental Health and Addiction (Trimbos Institute), Utrecht, Netherlands; ^7^Amsterdam Neuroscience, Amsterdam University Medical Center, Location Vrije Universiteit Medical Center, Amsterdam, Netherlands; ^8^Department of Anatomy and Neurosciences, Amsterdam University Medical Center, Location Vrije Universiteit Medical Center, Amsterdam, Netherlands

**Keywords:** PTSD, personality disorder, avoidant, dependent, obsessive-compulsive, cluster C, schema therapy, imagery rescripting

## Abstract

**Background:** Posttraumatic stress disorder (PTSD) is a serious and relatively common mental disorder causing a high burden of suffering. Whereas evidence-based treatments are available, dropout and non-response rates remain high. PTSD and Cluster C personality disorders (avoidant, dependent or obsessive-compulsive personality disorder; CPD) are highly comorbid and there is evidence for suboptimal treatment effects in this subgroup of patients. An integrated PTSD and CPD treatment may be needed to increase treatment efficacy. However, no studies directly comparing the efficacy of regular PTSD treatment and treatment tailored to PTSD and comorbid CPD are available. Whether integrated treatment is more effective than treatment focused on PTSD alone is important, since (1) no evidence-based guideline for PTSD and comorbid CPD treatment exists, and (2) treatment approaches to CPD are costly and time consuming. Present study design describes a randomized controlled trial (RCT) directly comparing trauma focused treatment with integrated trauma focused and personality focused treatment.

**Methods:** An RCT with two parallel groups design will be used to compare the clinical efficacy and cost-effectiveness of “standalone” imagery rescripting (*n* = 63) with integrated imagery rescripting and schema therapy (*n* = 63). This trial is part of a larger research project on PTSD and personality disorders. Predictors, mediators and outcome variables are measured at regular intervals over the course of 18 months. The main outcome is PTSD severity at 12 months. Additionally, machine-learning techniques will be used to predict treatment outcome using biopsychosocial variables.

**Discussion:** This study protocol outlines the first RCT aimed at directly comparing the clinical efficacy and cost-effectiveness of imagery rescripting and integrated imagery rescripting and schema therapy for treatment seeking adult patients with PTSD and comorbid cluster C personality pathology. Additionally, biopsychosocial variables will be used to predict treatment outcome. As such, the trial adds to the development of an empirically informed and individualized treatment indication process.

**Clinical Trial registration:** ClinicalTrials.gov, NCT03833531.

## Introduction

Posttraumatic stress disorder (PTSD) is a serious mental disorder characterized by intrusive symptoms, persistent avoidance, changes in cognition, affect, arousal and reactivity (1). These symptoms occur in response to exposure to (threat of) death, serious injury or sexual violation. The cross-national lifetime prevalence of PTSD in the general population is estimated at 3.9%. For those exposed to traumatic events the lifetime, 12-month and 30-day prevalence is estimated at 5.6, 2.8, and 1.4%, respectively (2), although this may depend on type of trauma exposure (3). Research shows that the burden of PTSD is high for both the individual (poor quality of life, chronic physical conditions) and society (4–7). Substantial empirical evidence supports the efficacy of (exposure-based) psychological treatments for PTSD (8–10). However, dropout and non-response rates for evidence-based PTSD treatments are high, both in real-world settings as well as in research trials (11–13). Recently, imagery rescripting (ImRs) has received attention as an evidence-based standalone treatment for posttraumatic stress disorder (14), although imagery techniques have been part of trauma-focused cognitive therapy for several decades (15). ImRs focuses on changing emotional and cognitive aspects of aversive memories by using imagination in order to facilitate reconsolidation of a less aversive memory of the actual event. In a meta-analysis, Morina et al. (16) conclude that ImRs holds promise as an effective and efficient intervention reducing psychological complaints associated with aversive memories. Furthermore, a recent study comparing ImRs and eye movement desensitization and reprocessing, a well-established evidence-based treatment for PTSD, showed that the two approaches were equally safe and effective in a population of patients with PTSD from childhood trauma (17).

Personality disorder (PD) comorbidity may be an important factor in high dropout and non-response rates in PTSD treatment. PDs are defined as inflexible, pervasive and pathological patterns of inner experiences and behavior in cognition, affect, interpersonal functioning and impulse control (1). High comorbidity exists between PTSD and PD (18). Whereas Van Minnen et al. (19) concluded that there is no evidence for higher dropout or lower treatment response in PTSD populations with high comorbidity (including PDs), other sources of evidence suggest that lower treatment effect is associated with childhood-onset trauma (10), childhood abuse related complex PTSD (20) and comorbid PDs (Snoek et al., under review).

Most research on PTSD and PD comorbidity is limited to PTSD and borderline PD (21), but there is evidence for high PTSD and CPD comorbidity as well (18). CPD includes avoidant, dependent and obsessive-compulsive PDs and is often labeled as the “anxious/fearful” cluster. Effective interventions for CPD include schema therapy (ST), an integrative psychotherapy for PDs (22–24). The scarcity of research on PTSD and CPD comorbidity is surprising given the high comorbidity (18) and the conceptual and empirical similarities and associations between PTSD and CPD found in the literature. First, childhood adversity is an important risk factor in both PTSD (25–30) and CPD (29, 31, 32). Second, emotion regulation difficulties in PTSD and CPD exhibit important similarities, such as underregulation of anxiety/fear, shame and guilt (33–36). Third, personality traits and coping styles such as harm avoidance, trait anxiety, behavioral and emotional inhibition, neuroticism and experiential avoidance have been found to be associated with both CPD and PTSD (37–46).

If research on PTSD and CPD comorbidity is scarce, research on treatment targeting this comorbidity is, to the best of the authors' knowledge, well-nigh non-existent. Such research is needed, because current best-practice treatment approaches to CPD and PTSD comorbidity differ, both in content and duration. For example, Ingenhoven (47) argues that treatment of complex PTSD and PDs should be aimed at stabilization instead of trauma focused work. Jongedijk et al. (48) note that only focusing on PTSD in those with high symptom severity and comorbidity may result in inadequate treatment selection. They state that focusing on personality and coping styles is important for those with severe PTSD who do not profit sufficiently from trauma focused therapy alone. By contrast, Van Minnen et al. (19, 49) argue that trauma focused treatment can and should be applied in the case of most if not all comorbidities, while recommending providing integrated or concurrent treatment of comorbid problems in the case of severe comorbidity. In support of this view, Markowitz et al. (50) found that PD diagnosis in a sample of 47 patients with PTSD and PD comorbidity often changed following trauma focused treatment. Indeed, most of the comorbid CPD were in remission posttreatment. This finding was replicated by Bovin et al. (51) in a longitudinal study on the course of PD characteristics after PTSD treatment in sample of 79 patients with PTSD and comorbid PD. Lastly, Dimaggio (52) and Wampold (53) presented an insightful account of the discussion about therapy efficacy, treatment integration and comorbidity in the case of PTSD, in which they argue that PTSD treatment selection could and should be a flexible process.

The debate about treatment selection in the case of comorbidity is further complicated by the fact that choice of treatment often depends on the primary diagnostic classification (e.g., trauma focused treatment in the case of PTSD and PD focused treatment in the case of CPD), which may be unclear in the case of comorbidity. Moreover, PTSD treatments are relatively brief, whereas CPD treatments such as ST tend to be much longer and more costly.

In sum, evidence-based PTSD treatments are available, CPD comorbidity may be an important factor explaining suboptimal treatment outcome, there is a lack of systematic research directly comparing trauma focused treatment with integrated trauma focused and PD focused treatment for PTSD and comorbid CPD and treatment selection in the case of comorbidity is problematic. The present study addresses these knowledge gaps by comparing treatment efficacy and cost-effectiveness of trauma focused treatment (ImRs) with integrated ImRs and PD focused treatment (ST). In a parallel trial, the treatment efficacy and cost-effectiveness of eye movement desensitization and reprocessing (EMDR) will be compared with integrated EMDR and dialectical behavior therapy in PTSD and comorbid borderline PD (54).

Whereas treatment efficacy studies are an important area of research, directly comparing two treatment approaches on a group level can only provide an answer to the question “what works in general” (55). Significant heterogeneity in treatment response will likely remain, even in the best performing treatments. In other words, whereas a treatment can be effective on a group level, it can be less effective, ineffective or even harmful on an individual or subgroup level. Therefore, identifying predictors and mediators of treatment effect, both on a group and individual is important to understand “what works for particular patients” (55). Several studies on prediction and mediation of psychological treatment outcome are available. Recently, machine-learning approaches have been employed to predict individual psychological treatment response in different populations (56–59) using biopsychosocial variables.

There is some evidence for genetic, neuronal and biomarker variables as predictors and mediators for PTSD treatment outcome. Examples of genetic and biomarker variables are the serotonin transporter polymorphism (5-HTTLPR), brain-derived neurotropic factor (BDNF), FK506 binding protein 5 (FKBP5), oxytocin and cortisol. Neuronal variables include activation patterns in parts of the salience network, such as the amygdala, anterior cingulate cortex and insula. Moreover, structural MRI studies identified anterior cingulate cortex, insula, amygdala and hippocampus volume as predictors of PTSD treatment outcome (60–63). On top of that, a multitude of candidate variables in PTSD research have been identified but not tested as predictors of treatment outcome (64, 65). In conclusion, whereas there is evidence for an association between various variables with PTSD, no single variable has proven to be a sufficient and specific predictor of PTSD and PTSD treatment outcome. Therefore, hypothesis-driven analysis of several key predictors is not sufficient and there is a need for a bottom-up, data-driven approach to predict the outcome of PTSD treatment.

In the present study, many candidate predictor variables will be measured before and after treatment (including, but not limited to, cortisol, FKBP5, 5HTTLPR, oxytocin, brain-derived neurotropic factor, numerous psychosocial variables and demographic variables). Machine-learning techniques will be used to identify relevant biopsychosocial variables to predict individual treatment response for those with PTSD and comorbid CPD. Moreover, structural and functional magnetic resonance imaging during rest and emotion processing and diffusion weighted imaging will be performed in a subgroup of patients. A detailed description of the neuroimaging part of the research project is presented separately (Aarts et al., under review).

## Objectives

### Primary Objectives

The primary objective of this study is to compare the treatment efficacy (reduction in PTSD severity posttreatment) of integrated ImRs and ST with ImRs only in treatment-seeking adults with PTSD and comorbid CPD. It is hypothesized that integrated ImRs and ST results in a higher effect size (*d* = 1.0) than ImRs only (*d* = 0.5).

### Secondary Objectives

The secondary objective of this study is to evaluate differences between ImRs only and ImRs and ST in terms of cost-effectiveness, treatment response and remission rates, PD symptom reduction and treatment dropout.

First, we hypothesize that integrated ImRs and ST is more cost-effective compared to ImRs only in terms of indirect medical and health-related costs.

Second, we hypothesize that there is a significant difference in number of treatment responders and PTSD remission rates.

Third, we hypothesize that PD symptom reduction is significantly larger in the integrated ImRs and ST condition than in the ImRs only condition. In addition, we hypothesize that PD symptom reduction (for both ImRs only and integrated ImRs and ST) is mediated by change in relevant schemas and schema modes.

Fourth, we hypothesize that ImRs and ST results in significantly lower dropout at T2 compared to ImRs only.

Fifth, we hypothesize that individual treatment response can be predicted above chance level using a combination of biological, demographic and psychosocial variables.

## Methods and Analysis

### Study Design

A single-blinded, randomized controlled superiority trial with two parallel groups design will be conducted at two locations of the Sinai Centrum, a community mental health institution located in Amstelveen and Amersfoort, the Netherlands. Data will be collected online (questionnaires) and on-site (interviews, hair samples). Blood samples will be collected at either of two local hospitals (Meander Medisch Centrum and Ziekenhuis Amstelland).

Patients will be randomized equally to ImRs only or integrated ImRs and ST. The study is embedded within a larger study project on treatment efficacy, working mechanisms and prediction of treatment outcome for those with PTSD and comorbid borderline PD or CPD (54, 66). The study protocol has been approved by the regional Medical Ethics Committee and will be conducted in accordance with the Dutch Medical Research Involving Human Subjects Act. The trial is registered under NCT03833531 on ClinicalTrials.gov (66).

### Participants and Recruitment

**Figure 1:**
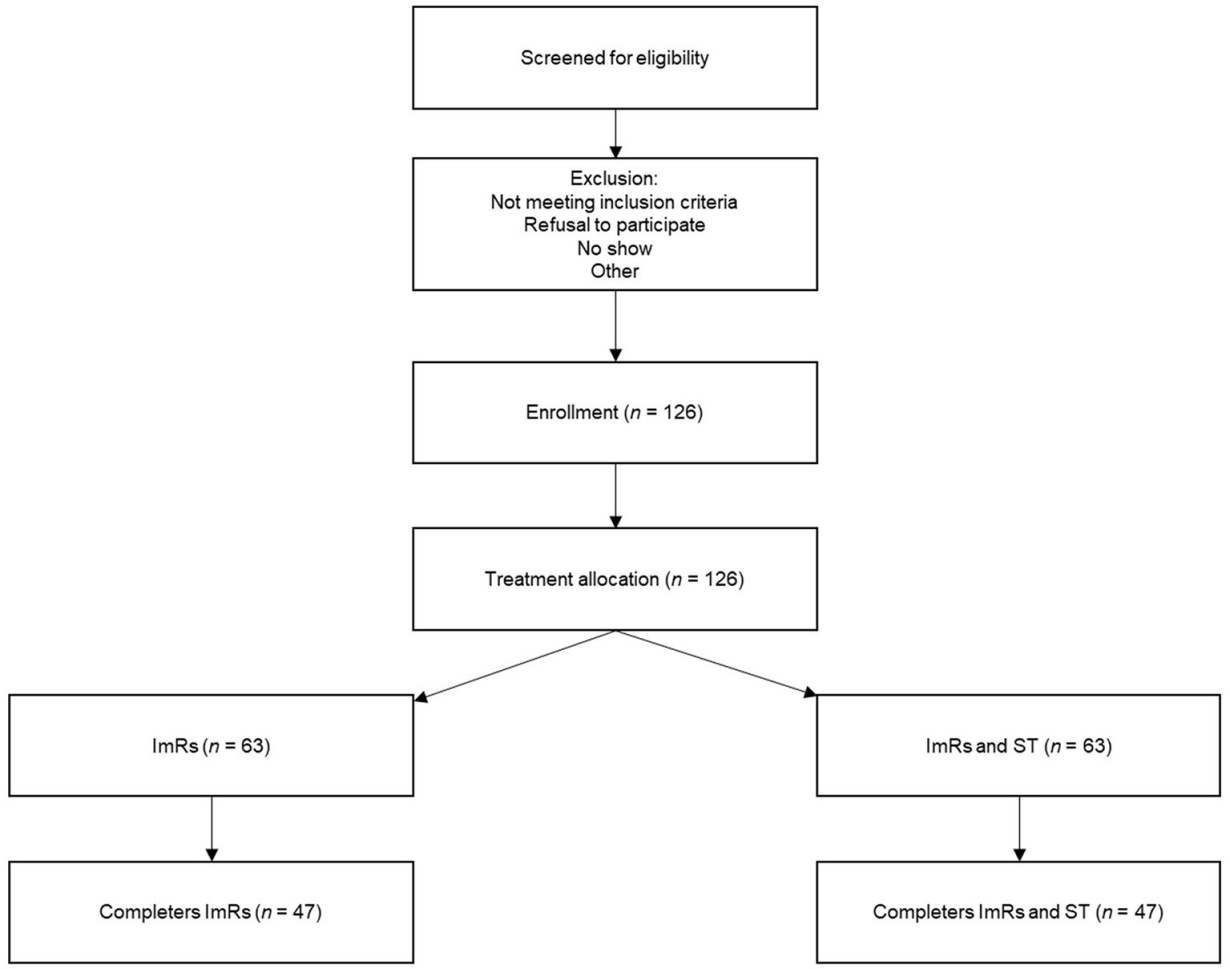
Patient flow diagram displaying the participant selection process and expected number of patients.

A patient flow diagram displaying the participant selection process and expected number of patients is presented in Figure 1. Participants will be treatment seeking adult patients presenting at Sinai Centrum, a mental health care institution specialized in treatment of trauma-related complaints. Patients will be screened for a main diagnosis of PTSD at intake and possible PD (SCID-5-SPQ). The intaker informs the patient about the trial, after which a research assistant contacts the patient. Patients who are excluded from the trial or refuse to participate will be offered regular trauma focused treatment.

Inclusion criteria are: (a) a primary diagnosis of PTSD according to DSM-5 criteria as measured by the Clinician Administered PTSD Scale—DSM-5 (67, 68); (b) a comorbid (sub)clinical avoidant, dependent and/or obsessive-compulsive PD, defined as at least the required number of DSM-5 criteria minus one as measured by the Structured Clinical Interview for DSM-5 Personality Disorders (SCID-5-P) (69, 70); (c) in the case of psychotropic medication use patients are required to have a stable medication regimen for at least 3 weeks prior to the start of the trial. Exclusion criteria are: (a) current psychosis; (b) comorbidity interfering with (group) treatment or randomization, such as severe outward aggression, treatment interfering substance and eating disorders, or treatment interfering somatic problems; (c) a primary diagnosis of paranoid, schizoid, schizotypal, narcissistic, histrionic or antisocial PDs; (d) IQ below 70; (e) insufficient mastery of the Dutch language for participation in group therapy.

### Sample Size

The sample size calculation is based on two-tailed *p* =0.05 significance testing, a power value of 80%, an estimated effect size of *d* = 0.5 for ImRs only and *d* = 1.0 for integrated ImRs and ST. Effect sizes are determined based on the expectancy that, on average, effect sizes of evidence-based PTSD treatments are *d* = 1.0 (8). It is expected that similar effects can be achieved for PTSD and comorbid CPD with integrated PTSD and PD treatment. To detect a minimal clinical relevant difference between treatments of *SD* = 0.5 on the Clinician Administered PTSD Scale for DSM-5 (CAPS-5) with an intra-person correlation coefficient of *r* =0.5, two follow-up measurements and 25% expected dropout a total sample size of approximately 126 patients is required (71). The 25% expected dropout rate is a (conservative) estimate based on Imel et al. (13).

### Procedure

**Figure 2:**
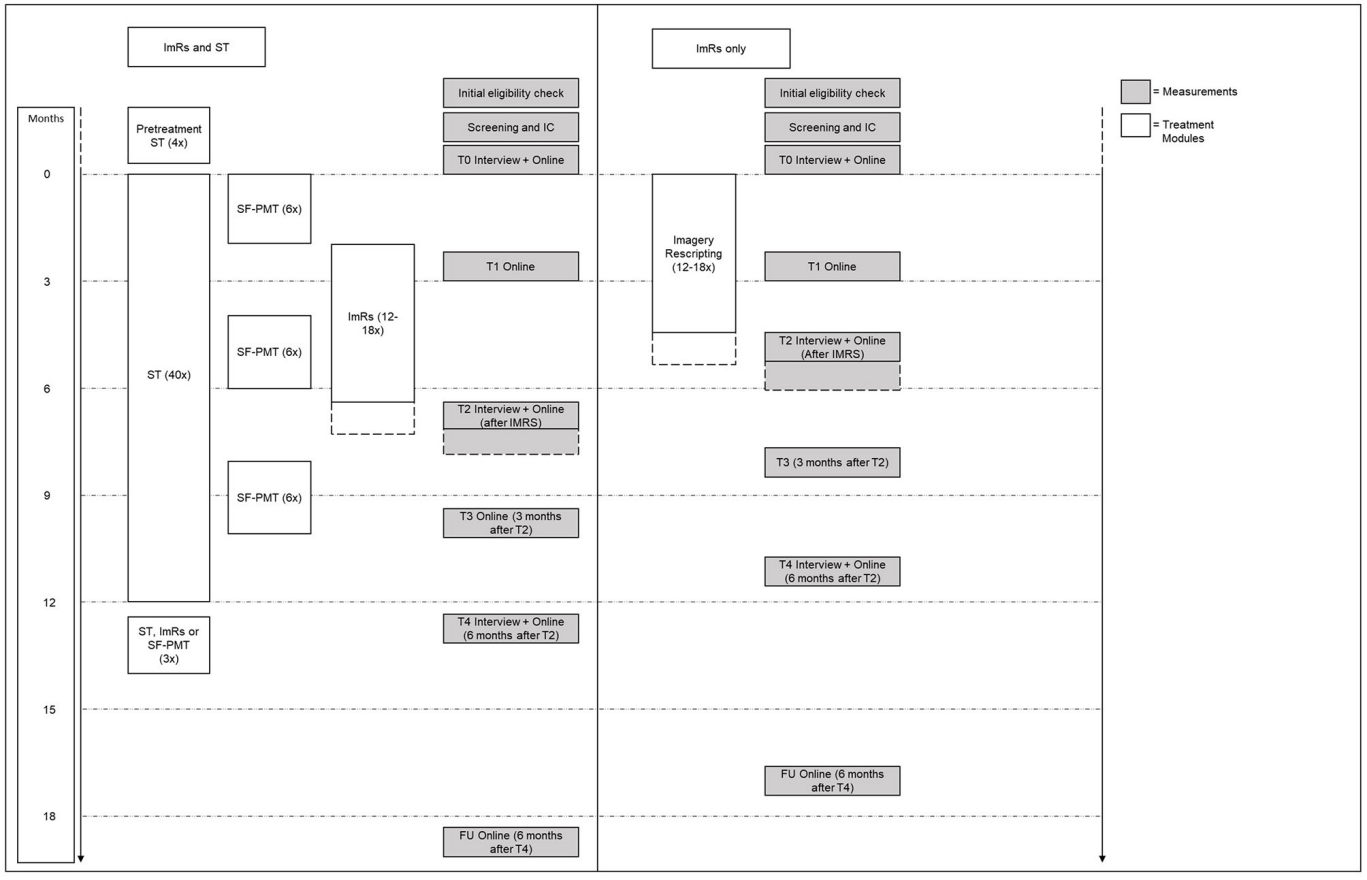
Overview of the trial procedure for ImRs only and integrated ImRs and ST including treatment and measurement timing. ImRs, imagery rescripting; ST, Schema therapy; SF-PMT, Schema focused psychomotor therapy; IC, Informed consent; FU, follow-up.

See Figure 2 for a flow chart of the trial procedure for both treatment groups. All interviews will be performed by trained doctoral level psychologists or students holding a BSc degree in clinical psychology working under supervision. Weekly meetings are held to ensure the quality of measurements (i.e., interrater reliability). Written informed consent (IC) will be obtained in person by one of the investigators after screening for eligibility using SCID-5-P and Structured Clinical Interview for DSM-5 (SCID-5-S) (72). Patients will receive detailed information about psychological treatment in the context of scientific research as part of the informed consent procedure. Next, patients are allocated to either ImRs only or integrated ImRs and ST by computer-generated block randomization (*n* = 4 per block). The allocation sequence is implemented through sequentially numbered, sealed envelopes prepared by an independent employee. The envelope is opened together with the patient by a research assistant not involved in research measurements in this trial. Blinding of trial participants and care providers is not possible due to the nature of psychotherapeutic interventions. Therefore, no unblinding procedures for revealing a participant's allocated intervention during the trial are needed.

Measurement interviews (screening, T0, T2, and T4) are conducted by research assistants blind to treatment allocation. Online self-report questionnaires will be filled out at every measurement occasion. See Table 1 for an overview of measurements for each time point. The timing of T2 is dependent on the end of ImRs as can be seen in Figure 2, both within treatment groups (T2 depends on number of ImRs sessions) as well as between treatment groups (start of ImRs differs between treatment groups). T3 and T4 take place 3 and 6 months after T2, respectively.

**Table 1:** Comprehensive list of measurements and time points.

**Measurement**	**Specification**	**Initial Eligibility**	**Screening**	**T0**	**T1**	**T2**	**T3**	**T4**	**FU**
PCL-5 (73)	PTSD Symptoms	x		x	x	x	x	x	x
SCID-5-SPQ (74)	Probable DSM-5 Personality Disorder	x							
OQ-45 (75, 76)	Psychiatric Symptoms	x		x	x	x	x	x	x
SCID-5-S (72)	DSM-5 Psychiatric Disorders		x					x	
SCID-5-P (69, 70)	DSM-5 Personality Disorder		x					x	
Body Measures	Height/Weight			x					
Blood Pressure	Systolic/Diastolic/Heart Rate			x					
Fasting Blood Sample	Biomarkers (5-HTTLPR, BDNF, FKBP-5, Oxytocin/OXTR, Full Blood Count)			x		x			
Hair Sample	Cortisol			x		x			
SST (77, 78)	Inhibitory Control			x					
N-back (79)	Working Memory (Capacity)			x					
CAPS-5 (67, 68)	DSM-5 PTSD			x		x		x	
Demographic Questionnaire	Demographic Variables			x					
LEC-5 (80)	Life Events	x		x					
CTQ (81, 82)	Childhood Trauma			x					
BDI-II (83, 84)	Depressive symptoms			x		x		x	
SZG (85)	Self-harm			x	x	x		x	x
DES-II (86)	Dissociative Symptoms			x		x		x	
DERS (87)	Emotion Regulation			x		x		x	
STAS (88, 89)	Anger			x		x		x	
PAI-BOR (90, 91)	Borderline Symptoms			x		x		x	
PSQI (92)	Sleep Quality			x		x		x	
AUDIT (93)	Unhealthy Alcohol Use			x		x		x	
RSQ (94)	Adult Attachment			x		x		x	
WHODAS 2.0 (95)	Health and Disability			x		x		x	x
CPQ-S (96)	Social Support			x		x		x	
EQ-5D-5L (97)	Generic Health Status								
Tic-P (98)									
WAV-12 (99, 100)	Working Alliance								
YSQ-75 (101)	Schemas			x		x		x	
SMI-118 (102)	Schema Modes			x		x		x	

*T0, baseline; T1, 3 months after baseline; T2, after ImRs; T3, 3 months after ImRs; T4, 6 months after ImRs; FU, follow-up; PCL-5, PTSD Checklist for DSM-5; SCID-5-SPQ, Structured Clinical Interview for DSM-5 Screening Personality Questionnaire; OQ-45, Outcome Questionnaire 45; SCID-5-S, Structured Clinical Interview for DSM-5; SCID-5-P, Structured Clinical Interview for DSM-5 Personality Disorders; SST, Stop-signal task; CAPS-5, Clinician Administered PTSD Scale for DSM-5; LEC-5, Life Events Checklist-5; CTQ, Childhood Trauma Questionnaire; BDI-II, Beck Depression Inventory-II; SZG, Screeningvragenlijst Opzettelijk Zelfverwondend Gedrag; DES-II, Dissociate Experiences Scale-II; DERS, Difficulties in Emotion Regulation; STAS, State Trait Anger Scale; PAI-BOR Personality Assessment Inventory-Borderline Features Scale; PSQI, Pittsburgh Sleep Quality Index; AUDIT, Alcohol Use Disorder Identification Test; RSQ, Relationship Scale Questionnaire; WHODAS 2.0, World Health Organization Disability Assessment Schedule=2.0; CPQ-S, Close Persons Questionnaire-Short; EQ-5D-5L, Five Level EuroQol Five Dimensions Health Questionnaire; TiC-P, Trimbos/iMTA questionnaire for costs associated with psychiatric illness; WAV-12, Werk Alliantie Vragenlijst-12; YQS-75, Young Schema Questionnaire-75; SMI-118, Schema Mode Inventory-118*.

In the case of treatment dropout, data collection is continued if possible. When patients refuse further extensive assessments, the CAPS-5, PTSD Checklist for DSM-5 (PCL-5) (73) and Outcome Questionnaire-45 (OQ-45) (75) are prioritized. Data collection is actively monitored by a research assistant, reminding patients to fill out questionnaires when appropriate.

Data will be entered in NetQ (www.netqhealthcare.nl) and stored on a secured server. Independent, double data entry and coding data with unique identification numbers and range checks for data values are part of data management procedures. Data collection, storage and sharing (including biological specimens) will be in accordance with the Dutch General Data Protection Regulation.

Patients are asked to discontinue any relevant concomitant psychological therapy for at least 12 months after start of treatment within the study trial. Relevance of concomitant care will be judged on an individual basis. Deviations from this rule (e.g., for reasons of emergency) will be monitored and registered. (Serious) adverse events will be carefully monitored and reported to the relevant authorities.

Finally, the need for post-trial care will be judged by a team of psychologists, nurses and psychiatrists post-treatment, after which patients will be referred to the appropriate care.

### Interventions

#### Schema Therapy

ST is an integrative psychotherapy combining elements of cognitive behavioral therapy, Gestalt therapy, attachment theory and psychodynamic theory (24). It is aimed at identifying and meeting core emotional needs that have not been met in early life. Two core concepts used in ST are schemas and schema modes. Schemas are temporally stable beliefs, feelings, sensations and thoughts shaping one's experience, whereas schema modes are defined as “those schemas or schema operations—adaptive or maladaptive—that are currently active for an individual” (24). There is some evidence for CPD-specific schema modes (103). ST is used in the treatment of PDs and other chronic disorders, including PTSD (104). There is increasing evidence for its efficacy in the treatment of PDs, including some evidence on CPD (22, 23, 105–108). More research on ST for CPD is currently being done (109).

The group ST protocol is based on Farrel, Reiss and Shaw (110) and elements of Vreeswijk, Broersen and Nadort (111) enriched with schema-focused psychomotor therapy (SF-PMT) and consists of four individual pretreatment sessions and 40 weekly 90-min sessions in an open group setting with two therapists and up to nine group members. After group therapy has ended, patients are offered three optional ‘booster’ sessions with a treatment module of choice (i.e., SF-PMT, ST or ImRs). Goal of the pretreatment sessions is to acquaint patients with the schema and schema mode concepts and to make an individualized case conceptualization. The group therapy is divided in three phases. In phase 1 (“preparation”) the emphasis lies on increasing group cohesion and identifying core schemas. Phase 2 (“change”) is dedicated to applying cognitive and experiential techniques, empathic confrontation, limited reparenting and group interactions. Core cognitive techniques include the use of flashcards, a schema diary, multidimensional evaluation, schema mode dialogues, role-play and behavioral experiments. Core experiential techniques include: imagery exercises such as safe place imagination, guided imagery, imagery through affect bridges and imagery rescripting, historical role-play and chair work. Both the cognitive and experiential techniques used in ST focus on identifying connections between past and present and are aimed at achieving change in the present.

Phase 3 is focused on the nearing end of the therapy phase. To promote the focus on experiential techniques as a core element of ST, 18 sessions of SF-PMT are added to the group therapy program. These sessions are divided over three modules consisting of 6 weekly sessions applied over 3 month intervals during the course of treatment (see Figure 2). The structure of the sessions follows the schema and schema mode models and consists of physical exercises designed to identify and experience maladaptive schemas and schema modes and promote healthy schemas and schema modes. The SF-PMT therapy protocol is based on Günther, Blokland-Vos, van Mook and Molenaar (112), Hoek and Scheffers (113) and Van der Meijden and Van der Meijden (114). See Figure 2 for an overview of the structure of ST and SF-PMT session planning.

#### Imagery Rescripting

ImRs, originally a technique used in cognitive behavioral therapy and ST, is increasingly being used as a standalone intervention for PTSD. The intervention consists of changing emotional and cognitive aspects of aversive memories (e.g., changing the catastrophic ending of a traumatic memory into a positive ending) by using imagination, thereby promoting reconsolidation of a less aversive memory of the actual event. ImRs as a standalone intervention for PTSD focuses on memories of physical and sexual abuse (i.e., the criterion A of PTSD), whereas in ImRs as a technique in ST the focus lies on aversive memories of (emotional) neglect. In a meta-analysis by Morina, Lancee and Arntz (16) it is concluded that ImRs is an effective and efficient intervention in treating aversive memories. Whereas ImRs is a less well-researched intervention in the treatment of PTSD compared to prolonged exposure and EMDR, it is chosen as the PTSD therapy in the present trial for perfect fit with the ST model, promoting treatment acceptability for the patient. The protocol is based on the protocol used in Raabe, Ehring, Marquenie, Olff and Kindt (14) and consists of twelve 75 min sessions administered within 14 weeks. Treatment is prolonged with a maximum of six 75 min sessions (administered within 6 months after therapy started) when indicated by a team of healthcare professionals.

The first session is dedicated to explaining the technique, constructing a trauma list guided by the PTSD criterion A. Whereas patients decide the order of the traumata themselves, they are advised to start with those traumata that occurred at childhood age. Finally, the ImRs technique will be practiced during this session targeting an adverse, but not traumatic, experience. Session 2 and 3 consist of imagery rescripting in which the therapist rescripts. The rescripting takes place in two phases. In phase 1 the patient is asked to recollect the traumatic event and to take on the perspective of their earlier self. The therapist then asks a series of questions aimed at amplifying vividness and emotionality of the image (i.e., “what do you see, hear, feel physically, feel emotionally, taste, what happens”). In phase 2 the therapist steps into the image and intervenes by averting the danger. The therapist can be creative in executing this, but it will usually consist of bringing the earlier version of the patient to safety, stopping the traumatic event from happening and punishing or banishing the assailant. This phase continues until the needs of the patient's earlier self have been met.

Patients start rescripting themselves from session 4–12, in three phases. Phase 1 is the same as when the therapist rescripts. In phase 2 the patient is instructed to enter the image as their present self. After a series of questions by the therapist aimed at amplifying vividness and emotionality of the patient's experience, the patient intervenes in the same way as when the therapist rescripts. In phase 3 the patient is again asked to take on the perspective of their earlier self and experience the rescripted situation. The therapist asks a series of questions to ensure activation of emotionality and vividness. Phase 2 and 3 are repeated until the needs of the patient's earlier self have been met.

Finally, if, after the practice session, participants are barely able to rescript themselves (e.g., because of severe dissociation or severe anxiety), the therapist will keep rescripting through sessions 4–6. In session 7–12 patients always rescript.

#### Therapists

All ImRs treatments will be administered by trained health care professionals (doctoral level psychologists in most cases) with ample experience in trauma-focused PTSD treatment, who received training in ImRs as a standalone intervention for PTSD. Additionally, therapists enrolled for ST will also be trained in ST. All therapists will participate in biweekly intervision and supervision sessions. Treatment sessions will be recorded on audio (IMRS) or video (ST). A random sample of these recordings will be rated on protocol adherence by the investigators. Moreover, therapists are required to fill out a short form after each session on which they can denote any particularities during the session. The number of enrolled therapists and their training background will be specified in subsequent publications.

### Outcomes

The primary outcome variable will be severity of PTSD as measured at three time points with the CAPS-5. Among secondary outcome variables are: (a) presence of PTSD (CAPS-5 diagnosis level) measured at three time points; (b) severity of PTSD as measured at five time points with the PCL-5; (c) presence of PD symptoms as measured with the SCID-5-P measured at two time points; (d) maladaptive schema and schema mode dimensional severity scores as measured with the Young Schema Questionnaire-75 (YSQ-75) (101) and Schema Mode Inventory-118 (SMI-118) (102), respectively; (e) treatment dropout; (f) cost-effectiveness estimated with the Treatment Inventory Cost—Psychiatric Patients (TiC-P) (98) and Five Level EuroQol Five Dimensions Health Questionnaire (EQ-5D-5L) (97).

### Assessments

See Table 1 for a comprehensive overview of all instruments used including references to studies on their psychometric properties, if available. Questionnaires used for inclusion and main outcome assessment are described below.

#### Clinician-Administered PTSD Scale for DSM-5

The CAPS-5 (67, 68) is a structured diagnostic interview for assessing DSM-5 PTSD. The 30-item interview provides dichotomous and continuous ratings of all 20 PTSD symptoms, duration, functional significance, dissociative symptoms and interview validity. Symptom severity scores are summed, resulting in a total severity score ranging from 0 to 80. Moreover, it contains an item prompting for a so-called “index trauma” (most distressing event). When a patient has difficulties selecting one particular index trauma, they are asked to select a category of trauma events (e.g., repeated child sexual abuse). Symptoms are rated over the past month. Each item is rated for frequency (number of times or percentage of time) and intensity (not present, minimal, clearly present, pronounced, extreme), resulting in a combined severity rating (0 = absent, 1 = mild/subthreshold, 2 = moderate/threshold, 3 = severe/markedly elevated, 4 = extreme). A symptom is counted as “present” if its severity rating is 2 or higher. Earlier versions of CAPS count as the gold standard to assess PTSD and initial research finds that convergent, discriminant validity and internal consistency, interrater reliability and test-retest reliability of the CAPS-5 are adequate (115, 116).

#### Structured Clinical Interview for DSM-5 (SCID-5-S)

The SCID-5-S (72) is the Dutch version of the SCID-5-Clinician Version (SCID-5-CV) (117), supplemented with parts of the SCID-5-Research Version (SCID-5-RV) (118). The SCID-5-S is a clinician-administered, semi-structured interview designed to systematically assess most DSM-5 disorders. It is widely used in clinical trials for its sound psychometric properties (119). SCID-5-S consists of 14 semi-independent modules to allow for selection of relevant sections. Relevant modules for present trial design are current depressive episode, manic episode, persistent depressive disorder, psychotic disorders, substance use disorders, panic disorder, agoraphobia, social anxiety disorder, specific phobia, generalized anxiety disorder, obsessive-compulsive disorder, anorexia nervosa, bulimia nervosa and avoidant/restrictive food intake disorder.

#### Structured Clinical Interview for DSM-5—Personality Disorders

The SCID-5-P (69, 70) is a semi-structured clinical interview considered the golden standard in systematically assessing DSM-5 PDs. Items represent DSM-5 PD criteria and are rated by a trained assessor on a 3-point scale (0 = absent, 1 = subclinical, 2 = present). The SCID-5-P features a 106-item self-report screening questionnaire (SCID-5-SPQ) (74), designed to screen for PDs. Items correspond with the initial SCID-5-P question and are rated with yes or no. SCID-5-SPQ results can be used to consecutively determine which PDs are further assessed by a clinician. The SCID-5-SPQ is designed to have a high false-positive rate and low false-negative rate, which has indeed been found for earlier versions in some studies (120–122).

Psychometric properties an earlier version of the SCID-5-P (i.e., SCID-II for DSM-IV) are satisfactory (123) and there is some preliminary evidence for adequate validity and reliability values for the SCID-5-P (124).

### Data Analysis

#### Primary Outcomes

Data will be analyzed on the basis of intention-to-treat analyses. Two-tailed significance levels are set at *p* = 0.05. Baseline variables will be examined separately for each condition using independent *t*-tests and chi-square tests. Significant confounding (*p* < 0.05, two-tailed) variables will be added as covariates in the statistical analyses.

The primary outcome variable will be severity of CAPS-5 PTSD measured at three time points. A multilevel model will be used to estimate the comparative efficacy of ImRs only vs. integrated ImRs and ST. If applicable, covariates and appropriate interaction terms will be added to the model.

A multilevel regression model is an appropriate technique to analyze nested data, in this case within-patient PTSD severity change (level 1: repeated measurements) and between-patient PTSD severity differences (level 2: type of treatment). Missing data for the outcome variable will be handled using multiple imputation (125).

#### Secondary Outcomes

##### Economic Evaluation

Economic evaluation (including a cost-effectiveness and cost-utility analysis) will be performed taking in account the CHEERS statement (126) and the 2015 ISPOR (127) guidelines on cost-effectiveness analysis for clinical trials. Costs will be estimated based on: direct (extracted from electronic patient file) and indirect (traveling, time spent) costs for the treatment program, additional healthcare use and productivity loss due to absenteeism and presenteeism. Cost estimates will be based on reference prices provided by Hakkaart-van Roijen et al. (128) and Kanters et al. (129). Costs for additional healthcare use will be estimated using the TiC-P. Effects will be based on CAPS-5 (cost-effectiveness) and Quality Adjusted Life Years (QALYs) derived from EQ-5D-5L (cost-utility). Based on costs and effects data, incremental cost-effectiveness ratios will be calculated and plotted on cost-effectiveness planes. We will also prepare cost-effectiveness acceptability curves to present the cost-effectiveness of the experimental intervention compared to the control condition at varying willingness-to-pay (WTP) levels. Finally, WTP analysis will be performed to compare the incremental costs per incremental QALY to the common Dutch QALY WTP thresholds. In the base case scenario, cost-effectiveness and cost-utility analyses will be performed from the societal perspective on the basis of intention-to-treat with a time-horizon of approximately 18 months after T0 (i.e., at FU). Bootstrapping methods and sensitivity analyses will be performed to estimate stochastic uncertainty and evaluate robustness of findings.

##### Treatment Response and Remission

Treatment response is defined as a PTSD severity change score of pooled SD ≥1.0 between baseline and T4 measured with CAPS-5 based on the mean effect size of PTSD treatment reported by Cusack et al. (8). Remission is defined as failure to reach the DSM-5 PTSD criteria threshold at T4. Between-group differences in treatment responder status (0 = non-responder, 1 = responder) will be analyzed using generalized mixed modeling with a binomial link function. Furthermore, an additional analysis is performed with treatment responder status defined as PTSD status (0 = yes, 1 = no).

##### PD Symptoms

SCID-5-P symptom change after treatment will be analyzed using a multilevel model using the dimensional score of all CPDs combined as outcome variable and treatment type as categorical predictor variable.

##### Dropout

Treatment dropout rates for both conditions will be analyzed using survival analysis.

##### Mediation

Relevant schemas and schema modes (based on literature) will be analyzed as mediators of PD symptom change, for example using structural equation modeling.

##### Prediction

Prediction analyses will be performed using a machine-learning approach (e.g., random forest classification). The outcome variable will be treatment responder status (0 = non-responder, 1 = responder). A selection of the most predictive variables will be included in a final model. See Schmitgen et al. (56) for a recent example of this approach in an RCT.

## Discussion

To the best of the author's knowledge, this study will be the first to directly compare a trauma focused treatment (ImRs only) vs. integrated trauma focused and PD focused treatment (ImRs and ST) in a sample of treatment seeking adult patients with PTSD and comorbid CPD. It is hypothesized that ImRs and ST is more efficacious than ImRs only, both from a clinical as well as from an economic cost-effectiveness point of view. Another important, exploratory aim of the study will be to predict treatment outcome using baseline biopsychosocial individual difference variables.

The present study adds to the development of an empirically informed and individualized treatment indication process and a more efficient dissemination of scarce mental health funds. As such, the present study holds great potential for clinical practice. Additionally, the study will be performed in a mental health institution specialized in treatment of trauma related psychopathology. The naturalistic setting, in combination with a patient population often identified as difficult-to-treat, makes this study relatively robust to known criticisms of RCTs, such as RCTs being too strict on inclusion and exclusion criteria, resulting in unrealistically homogeneous patient groups, thereby limiting generalizability of findings. Finally, CPD is an understudied category of mental disorder (130–132). Present study will be an important contribution to the study of CPD.

However, the results of the trial must be interpreted in the light of several potential limitations. First, two possible limitations arising from current study design are that (a) both therapy duration as well as therapy dosage differ between conditions and (b) as a consequence, the timing of measurements differs between conditions. This comparison is important, because whereas an alternative would balance duration and dosage of treatment between conditions – for example by adding treatment as usual – no guidelines on concurrent treatment as usual next to trauma focused treatment for this specific comorbid population exists. In fact, providing trauma focused treatment only is the status quo and, in essence, can be considered treatment as usual. Therefore, comparing integrated treatment with trauma focused treatment only provides a naturalistically valid way of assessing the added effect of PD focused treatment to treatment as usual, at the cost of internal validity. Furthermore, the problem introduced by variability in timing of measurements (both within as well as between groups, see Figure 2) is to be preferred over planning measurements using fixed time points, because in the latter scenario error variance due to variability in timing of measurements since the end of treatment is introduced. It is our belief that measuring as close to the end of treatment as possible is the most valid way of assessing treatment effects.

Second, while we hypothesize that dropout will be lower in the ImRs and ST condition for substantive reasons, group therapy may be challenging for patients suffering from CPDs. For example, those with avoidant PD tend to avoid disclosing themselves in a group and react to feelings of anxiety with behavioral avoidance (in this case, avoiding group therapy). However, we expect that CPD comorbidity is a factor explaining dropout and non-response in PTSD only treatments. Adding therapy targeting the very avoidance behavior otherwise causing dropout from PTSD treatment may, in fact, *prevent* them from dropping out. To minimize attrition risk patients are carefully and repeatedly informed about the specifics of their treatment program.

Third, the study is powered as a superiority trial. Thus, when the between-group effect size is *SD* < 0.5, it cannot be concluded that ImRs only is equivalent to ImRs and ST. However, in that scenario it can be concluded that a clinically meaningful or economically efficient difference could not be demonstrated.

Fourth, although the aim is to study severe psychopathology, some patients with only subclinical PDs (i.e., cut-off score minus one) will also be included in the present study for several reasons. PDs often co-occur with other PDs (133–135). Therefore, significant but subclinical scores on one DSM-5 PD category can be expected to be accompanied by (sub)clinical scores on at least one other DSM-5 PD category. Indeed, it is expected that most if not all included patients would satisfy criteria for other specified PD when using a cut-off score of 5 diagnostic criteria suggested by Verheul, Bartak and Widiger (136). This approach is in line with recent insights that categorical descriptions are empirically poor descriptors of psychopathology and a dimensional approach is more fruitful both for research as well as for clinical practice (135, 137). Thus, while present trial will use discrete categories—as this is still the most common method for patient inclusion in research and facilitates comparisons between trials—the inclusion threshold is lowered by one symptom to minimize false negatives for categorical subclinical but dimensional significant personality pathology. Moreover, personality pathology will be carefully distinguished from trauma related complaints in the present study in SCID-5-P interviews and through weekly intervision meetings. Lastly, SCID-5-P items often do not yield sufficient information on the general criteria of a PD (1). A PD must consist of maladaptive patterns of cognitions, affect, interpersonal functioning and impulse control that are pathological, temporally stable and pervasive across life domains. For present study, these criteria are ascertained on an item-by-item basis. This may lead to stricter diagnostic decision rules than other studies assessing PDs.

Fifth, the present study is conducted parallel to another RCT on PTSD with borderline PD comorbidity. Both diagnostic groups receive a different trauma focused and PD focused treatment. This precludes direct comparisons of treatment-specific efficacy between diagnostic groups. While this is not a limitation *per se* (the aim of the RCT is not to compare diagnostic groups), a considerable comorbidity between CPD and borderline PD is expected (138). In the current design, some patients with both significant borderline PD and CPD will be classified as inclusion for the comorbid borderline PD trial, while some patients with significant borderline PD and CPD will be classified as inclusion for the comorbid CPD trial. The study is not powered to compare ImRs and/or ST vs. EMDR and/or DBT for this group of patients. Therefore, any *post hoc* analyses on this subgroup of patients will be explorative.

Sixth, treatment length and intensity differs considerably between conditions. Therefore, differences in efficacy between conditions may in part be attributable to differences in therapy dosage. However, even a more intensive and hence more costly treatment may be cost-effective compared to the much shorter, less costly treatment. Therefore, cost-effectiveness analyses are an important part of the present study design.

In sum, this randomized controlled trial will be the first direct comparison between trauma focused treatment and integrated trauma focused and PD focused treatment for treatment seeking, adult patients with PTSD and comorbid CPD. It addresses an important knowledge gap in the literature and has the potential to be of great value to clinical practice by adding to the knowledge of what works for individual patients in a complex, often characterized as difficult-to-treat population.

## Ethics and Dissemination

The study will be performed in accordance with the Declaration of Helsinki and the International Conference on Harmonization—Good Clinical Practice guidelines. The study protocol was approved by the regional medical ethics committee (METC; registration number A2018.428(2017.335). The METC will be updated about any (non)substantial amendments. A summary of the study progress will be submitted once a year to the accredited Ethical Review Board.

Informed consent is obtained before any study-specific procedures take place. After explanation of the aims, methods, benefits and potential hazards of the study (including randomization to treatment), informed consent is obtained by the investigator. Patients are informed that they are free to refuse to participate in the study, or that they can withdraw their consent at any time without having to specify the reason and without incurring any penalty or withholding of treatment on the part of the investigator. Only patients who are able to give legal consent will be entered into the study. Signed informed consents are filed by the investigator.

Participants will be informed about the trial results. Study results, including primary and secondary outcomes, economic evaluation and prediction analyses will be reported and submitted for publication in scientific, peer-reviewed journals. Authors will participate in (inter)national conferences to facilitate communication of results. Participation of the authors in future publications associated with the present study is intended.

## Ethics Statement

The studies involving human participants were reviewed and approved by Medisch-ethische toetsingscommissie VU medisch centrum/Medical Ethical Committee VU Medical Center. The patients/participants provided their written informed consent to participate in this study.

## Author Contributions

AE, JD, AB, IA, AS, MB, CV, OH, and KT contributed to the design of the study. AE wrote the first draft of the manuscript under supervision of JD, AB, and KT. JD, AB, IA, AS, MB, CV, OH, and KT contributed to the final version of the manuscript by providing AE with feedback. All authors contributed to the article and approved the submitted version.

## Conflict of Interest

The authors declare that the research was conducted in the absence of any commercial or financial relationships that could be construed as a potential conflict of interest.
